# The impact of nurses' sense of security on turnover intention during the normalization of COVID-19 epidemic: The mediating role of work engagement

**DOI:** 10.3389/fpubh.2022.1051895

**Published:** 2022-12-01

**Authors:** Yao Tang, Luís M. Dias Martins, Shi-bin Wang, Qing-xia He, Hui-gen Huang

**Affiliations:** ^1^Guangdong Mental Health Center, Guangdong Provincial People's Hospital, Guangdong Academy of Medical Sciences, Guangzhou, China; ^2^Department of Nursing, Shantou University Medical College, Shantou, China; ^3^BRU-Business Research Unit, ISCTE-IUL (Institute University of Lisbon), Lisbon, Portugal; ^4^Nursing Department, Zhuhai People's Hospital, Zhuhai, China; ^5^School of Health Management, Southern Medical University, Guangzhou, China

**Keywords:** COVID-19, normalization, sense of security, work engagement, turnover intention, nurse

## Abstract

**Background:**

COVID-19 pandemic has entered a normal stage in China. During this phase, nurses have an increased workload and mental health issues that threaten the sense of security. Poor sense of security may have a considerable impact on turnover intention through low work engagement. It was challenging to maintain the nurse workforce. Fewer studies have been conducted on the effect of nurses' sense of security on their turnover intention in that phase. This study aimed to investigate the interrelationship between nurses' sense of security, work engagement, and turnover intention during the normalization phase of the epidemic in China and to explore the impact of sense of security on turnover intention.

**Methods:**

A cross-sectional survey was conducted from September 2020 to May 2021 in Guangdong Province, China. Data were collected online using Sense of Security Scale for Medical Staff (SSS-MS), Utrecht Work Engagement Scale (UWES), and Turnover Intention Scale. Pearson's correlation analysis was used to assess the correlation between sense of security, work engagement, and turnover intention. The hypothesis model used multiple linear regression models and the bootstrapping procedure to analyze the relationship between these variables.

**Results:**

Data were collected from 2,480 nurses who met the inclusion criteria. Over half(64.5%) of nurses had a high and very high turnover intention. After controlling the demographic and working variables, sense of security (β = 0.291, *P* < 0.001) had a direct positive effect on work engagement. Sense of security (β = −0.447, *P* < 0.001) and work engagement (β = −0.484, *P* < 0.001) had a direct negative effect on turnover intention. Sense of security and all of its components were associated with turnover intention through the partially mediating effects of work engagement.

**Conclusions:**

Nurses' turnover intention was at a high level during the normalization phase of the epidemic. Sense of security and its components act as positive resources to reduce turnover intention by improving work engagement. Policy makers and managers may pay attention to the needs of nurses' sense of security, which may be a new perspective to help managers reduce their turnover intention and stabilize the nurse team.

## Introduction

Nurses, as the largest health care professional grouping, play a critically significant role in the fight against the COVID-19 epidemic. The shortage of nurses in China remains a severe problem, and nurse turnover exacerbates shortages. Although the number of nurses in China is increasing yearly, the shortage of nurses due to the vast population base is a problem that cannot be ignored. According to the latest the latest WHO data (WHO NHWA Data Platform–January 2021 update), the density of nurses and midwives per 1,000 population in Europe and the Americas Region exceeds 8, and the average density of nurses and midwives per 1,000 population in 194 countries is 4.49 (1). However, according to the statistical bulletin on China's health care development in 2021 released by the China Health Care Commission, there were only 3.56 registered nurses per 1,000 population (2). Thus, the shortage of nurses has become a major health human resource problem to be solved urgently in China.

At the end of April 2020, China entered the phase of normalization of COVID-19 epidemic prevention and control. The goal was to strictly prevent imported cases, center on nucleic acid testing for risk prevention and control, and control the spread of the epidemic within 2 to 3 incubation periods (3). The constant mutation of the severe acute respiratory syndrome coronavirus 2 (SARS-CoV-2) poses a considerable challenge to outbreak prevention and control (4). Although the epidemic was not spreading or outbreaking on a large scale, unanticipated sporadic disseminated cases occurred from time to time. The work of many nurses has changed dramatically from the past. Due to the shortage of human resources in the community, nurses in general hospitals have been required to accept out-of-hospital nursing assignments in addition to clinical care in the hospital. For example, they were sent to the community or other places to participate in nucleic acid testing and vaccination (5). Moreover, the change in workflow and shift patterns have also resulted in nurses working long hours, insufficient rest, and constant overtime (6). The need for nurses in China has continued to increase during the normalization phase of the COVID-19 epidemic.

The COVID-19 epidemic contributed to an adverse working environment for healthcare workers. In the face of increased risk of infection, long work shifts, overload, and lifestyle changes may significantly affect healthcare workers' mental health, such as burnout, stress, depression and anxiety, insomnia, post-traumatic stress disorder, and insomnia (7, 8). Results from meta-studies during the COVID-19 epidemic reported that nurses demonstrated significantly worse mental health outcomes compared to other healthcare workers (9, 10). Since most nurses are women, they are more sensitive to the perceived emotional burden in the workplace (11). Moreover, the occupational characteristics of nurses tend to more extended patient contact and follow-up, experiencing more workload and risk of infection (12, 13). Nurses also have a negative psychological impact during the normalization phase of the COVID-19 epidemic (14). Increasing work stress and psychological stress made some of them opt to leave, but not in nursing, leading to an increased shortage (15). The shortage may aggravate the burden on the healthcare system (16).

Turnover intention is an employee's behavioral intention or attitude toward leaving his or her organization or unit, which is a psychological state or tendency before generating turnover behavior (17). Moreover, it is a crucial antecedent variable for turnover behavior and can predict turnover behavior (18). Research has previously observed that nurse turnover impacts the quality of care and generates many lost economic benefits (19), rendering it an ongoing concern for administrators. Notably, nurses with higher turnover intention may not decide to engage in turnover behavior. However, high turnover intention were associated with absenteeism (20) and missed nursing care (21). Thus, nurses' willingness to work may affect patients and their self-safety, especially caring for COVID-19 patients. Surveys such as that conducted by Nashwan have shown that nurses' turnover intention increased significantly during COVID-19 (from a mean score of 13.24 to 15.54) (22). Therefore, the turnover intention of nurses under the normalization of the epidemic situation needs further research.

Previous studies have mentioned job insecurity as a predictor of turnover (23). During COVID-19, many studies verified that insecurity was related to turnover intention (24–26). Insecurity is associated with physical and mental health well-being, and insecurity can contribute to the development of mental illness and somatic symptoms, and job burnout (27–29). However, due to medical professionals' professional and workplace characteristics, their security may be derived from their experiences at work, such as social media, doctor-patient relationships, medical work environment, and promotion system (30, 31). According to the work characteristics of medical personnel, the sense of security of medical personnel consists of five components: work environment, patient, self-competence, organizational management, and social support (32). During the normalization of the COVID-19 epidemic, overload and nurse-patient conflicts also lead to a high incidence of anxiety and depression among nurses (33, 34). These psychological risks may threaten the nurse's sense of security during this period (35), and the poor sense of security may increase the possibility of turnover. The association between insecurity and turnover intention among hotel employees (36) and corporate employees (37) during the COVID-19 pandemic has been identified. Nevertheless, has been scarce research on the relationship between nurse sense of security and turnover intention during the normalization of the COVID-19 epidemic.

Work engagement was defined as a positive, fulfilling psychological state characterized by vigor (i.e., abundant energy and resilience); dedication (i.e., a sense of meaning, pride, and challenge to the work); absorption (i.e., concentration on the work and readily absorbed in work) (38). Since the rising of positive psychology, work engagement has received more and more attention. Previous studies have referred to work engagement as a protective factor for turnover intention (39), which promotes job outcomes such as job satisfaction, job performance, and organizational citizenship behavior (40, 41).

**Figure 1:**
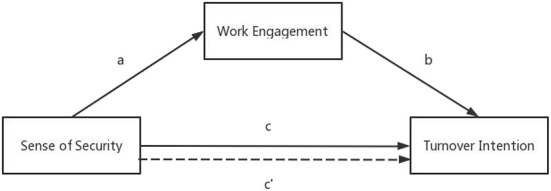
The theoretical model.

The research was based on the job demands-resources model (JD-R Model) and the conservation of resources (COR) theory. The characteristics of any job could be divided into job demands and resources concerning the JD-R model (42). Job demands refer to characteristics that require continuous physical or mental consumption. In contrast, job resources refer to characteristics that can support and help employees, reduce consumption, and motivate growth. Sense of security and work engagement help prevent burnout (43, 44). They belong to positive job resources. According to the positive process of the JD-R model, work engagement plays a vital role in acting as a bridge between available job resources and positive work outcomes (i.e., reduced turnover intention). An adequate sense of security could enable this positive path to reduce turnover intention by increasing work engagement. COR theory suggests that individuals tend to avoid the loss of resources (i.e., they have a tendency to retain, protect, and create resources) (45). Sense of security and work engagement are significant job resources that individuals value. Thus, avoiding the loss of resources may lead individuals to choose to stay with the organization. Based on the above, this study proposes a conceptual model map, as shown in Figure 1. In addition, studies showed a correlation between sense of security and turnover intention. In this mediation model, however, we are unclear whether the components of sense of security contribute to it. Therefore, we considered different components of sense of security to bring into this research model to fill this gap in research.

The aim of this study is to examine the relationship between sense of security, work engagement, and turnover intention among Chinese nurses under the epidemic normalization of the COVID-19 epidemic. We propose four hypotheses as follows:

H1: Sense of security has a positive impact on work engagement (path a).

H2: Sense of security has a negative impact on turnover intention (path c).

H3: Work engagement negatively has a negative impact on turnover intention (path b).

H4: The effects of sense of security on turnover intention are partially mediated by work engagement (path c′).

## Materials and methods

### Design, setting and participants

A cross-sectional study was conducted between September 2020 and May 2021 using snowball and convenience sampling in Guangdong Province, China. The sample size was estimated based on Wu's (46) proposal that a sample size >200 is needed to construct a stable model. Therefore, we aimed to collect at least 200 valid questionnaires. Inclusion criteria: (1) Age ≥18, engaged in clinical work; (2) No mental or cognitive dysfunction; (3) Registered nurses who are qualified to practice; (4) Volunteer to participate in this study. Exclusion criteria: (1) Practice nurses and trainee nurses who have not obtained the practice qualification; (2) Nurses who did not work in the clinic during the survey period (such as studying abroad, not participating in the clinic while engaged in management work, asking for leave and being hospitalized).

### Data collection

We sent the questionnaire to the members of the Guangdong Nursing Association through Wenjuanxing (a professional questionnaire website, https://www.wjx.cn). We explained the aim of the study and the principles of voluntary and anonymous participation. Then, the members forwarded the questionnaire to the head nurse of each nursing unit, who invited the nurses in each department to fill in the questionnaire.

### Ethical considerations

The Ethics Committee has approved this study of Guangdong Provincial People's Hospital (NO. Ky2020-579-01). On the premise of strictly implementing the informed consent and voluntary principles, the research objectives, methods and significance was explained to the subjects on the title page of the questionnaire, and consent was obtained before the questionnaire could be filled out. We fully respect subjects' informed consent and privacy, and subjects can withdraw from the investigation to comply with their wishes.

### Measurements

#### Demographic characteristics and working characteristics

We designed the demographic and working information questionnaire, including nine items: gender, age, marital status, education, professional title, work seniority, hospital level, employment status, and department. Based on past research on turnover intention (47–49), we set these variables as covariates to exclude potential confounding bias in examining mediation effects.

### Sense of security

Sense of security was measured using the Sense of Security Scale for Medical Staff (SSS-MS), validated among the Chinese medical staff (32). The scale was mainly composed of 5 dimensions and 22 items: environmental (4 items; e.g., “I am worried about the physical environment and occupational exposure in the hospital”;α = 0.843), patient (4 items; e.g., “The patient's distrust scares me at work”;α = 0.890), self (3 items; e.g., “I am worried that the lack of comprehensive ability leads to contradictions and inability to deal with emergencies”;α = 0.788), organizational management (7 items; e.g., “The lack of care and support from my superiors made me feel isolated and helpless”;α = 0.917), and social support (4 items; e.g., “The tense atmosphere of medical treatment reported by media reports upsets me”;α = 0.890). The scale was scored using the Likert 5-level method, ranging from 1 (absolutely agree) to 5 (absolutely disagree). The higher the score, the higher the sense of security. The scale has been shown to have good reliability among medical personnel. We calculated the average and the total scale score (α = 0.966).

### Work engagement

Work engagement was measured by the Chinese version of the Utrecht Work Engagement Scale (UWES) (50). The scale contained 3 dimensions and 16-item, vigor (6 items; e.g., “I feel strong and vigorous at work”;α = 0.902), dedication(5 items; e.g., “I am proud of my current job”;α = 0.953), and absorption (5 items; e.g., “When I am working all I think about is work”;α = 0.907). The scale was scored using the Likert 7-level method, ranging from 0 (never) to 6 (always). The higher the score, the greater the work engagement. We calculated the average and total scale scores (α = 0.962).

### Turnover intention

To assess turnover intention, we used the Turnover Intention Scale (TI), translated and revised by LI Dongrong and LI Jingyuan (49). This scale consisted of 6 items under three subscales: TII-the possibility of turnover (2 items; e.g., I am going to quit my present job'); TIII-the motivation to look for another job (2 items; e.g., I want to find another job of the same nature); TIIII-the possibility of obtaining another job (2 items; e.g., 'The likelihood that I will find a suitable position in another organization). The scale was scored using the Likert 4-level method, ranging from 1 (absolutely disagree) to 4 (absolutely agree). The higher the score, the greater the turnover intention. The higher the score, the greater the turnover intention. Nurses' turnover intention was classified as very low turnover intention ( ≤ 1), low turnover intention (>1, ≤ 2), high turnover intention (>2, ≤ 3), and very high turnover intention (>3). We calculated the average and total scale scores (α = 0.799).

### Data analysis

IBM SPSS 26.0 and AMOS 24.0 were used for analysis. First, the data with the normal distribution of continuous variables were represented by mean and standard deviation. The categorical variables were described as frequency and percentage. Secondly, Pearson's correlation analysis was used to analyze the relationship between sense of security, work engagement, and turnover intention for normally distributed data. Finally, we used the PROCESS macro mediation model (model 4) (51) in SPSS. We set the sense of security as the independent variable, work engagement as the intermediary variable, and turnover intention as the dependent variable to estimate the size and significance of the mediation effect. The bootstrap method was used, with a sample size of 5,000 and a confidence interval of 95%. The mediation effect was considered significant if the confidence interval did not include zero. The same procedure was conducted to analyze five dimensions of sense of security to determine different components' effects (52).

## Results

### Participants characteristics

A total of 2,554 nurses were distributed the online questionnaire, and 2,480 returned the survey (response rate: 97.1%). Participants were from 29 hospitals in Guangdong Province, including 14 tertiary hospitals, 8 secondary hospitals, and 7 primary hospitals. Most of them (89.9%) were female,51.5% were < 30 years old,60.0% were married,61.8% had junior college education,71.9% held the junior professional title, 34.0% had worked in hospitals for 6–10 years, 59.0% had worked in tertiary hospitals, and 64.6% were temporary. 26.5% of them were from the medicine unit. The characteristics of other participants are shown in Table 1.

**Table 1:** Demographic characteristics of participants(*N* = 2480).

**Variable**	**Categories**	***n*(%)**
Gender	Male	250(10.1)
	Female	2230(89.9)
Age (years)	≤ 30	1277(51.5)
	31-40	835(33.7)
	≥41	368(14.8)
Years of service (years)	≤ 5	746(30.1)
	6–10	844(34.0)
	11–20	555(22.4)
	≥21	335(13.5)
Professional title	Junior	1782(71.9)
	Intermediate	602(24.3)
	Senior	96(3.9)
Marital status	Married	1489(60.0)
	Unmarried	962(38.8)
	Divorce or widowed	29(1.2)
Education level	Secondary school	18(0.7)
	Junior college	1532(61.8)
	Undergraduate or above	930(37.5)
Department	Intensive care unit	173(7.0)
	Medicine	656(26.5)
	Surgery	473(19.1)
	Obstetrics or gynecology	298(12.0)
	Pediatrics	173(7.0)
	Emergency	264(10.6)
	Medical technology department	281(6.5)
	Others	162(17.8)
Employment status	Temporary	1603(64.6)
	Permanent	877(35.4)
Hospital level	Primary hospital	178(7.2)
	Secondary hospital	840(33.9)
	Tertiary hospital	1462(59.0)
Turnover intention	Very low turnover intention	73(2.9)
	Low turnover intention	817(32.9)
	High turnover intention	1147(46.3)
	Very high turnover intention	443(17.9)

### Descriptions and correlations of variables

Pearson's correlation coefficients of sense of security, turnover intention, and work engagement are shown in Table 2. Sense of security was positively correlated with work engagement. Turnover intention was negatively related to sense of security and work engagement.

**Table 2:** Means, standard deviations (SD) and correlations among study variables (*N* = 2480).

	**Variables**	**Mean**	**SD**	**1**	**2**	**3**
1	Sense of security	79.62	19.61	1		
2	Work engagement	65.76	20.24	0.260***	1	
3	Turnover intention	14.13	4.28	−0.390***	−0.413***	1

***p < 0.001.

### Test of the hypothesized model and associations among variable

Table 3 tabulates the results of the regression analyses examining the mediation hypothesis. The negative effect of sense of security on turnover intention was significant (β = −0.447, t = −24.522, *p* < 0.001), and the indirect negative effect of sense of security on turnover intention remained significant (β = −0.306, t = −18.923, *P* < 0.001) when the mediating variables were involved. Sense of security had a significant positive impact effect on work engagement (β = 0.291, t = 14.706, *P* < 0.001), and work engagement had a significant negative predictive effect on turnover intention (β = −0.484, t = −30.539, *P* < 0.001). As shown in Table 4, the association between sense of security and turnover intention was partly mediated by work engagement. As to its five dimensions, the relationship between environment, patient, self, organizational management, social support, and turnover intention was also partially mediated by work engagement.

**Table 3:** Results of the regression models testing the mediation hypothesis (*N* = 2480).

**Variable**	**Total effect of sense of security on turnover intention**		**Direct effect of sense of security on work engagement**		**Direct effects of sense of security on turnover intention and work engagement on turnover intention**	
	Path c		Path a		Path c' and Path b	
	**β**	**t**	**β**	**t**	**β**	**t**
Sense of security	−0.447	−24.522***	0.291	14.706***	−0.306	−18.923***
Work engagement	–		–		−0.484	−30.539***
Gender						
Male (ref)						
Female	0.021	1.175	0.023	1.165	0.033	2.097*
Age (years)						
≤ 30 (ref)						
31–40	−0.031	−1.144	0.066	2.253*	0.001	0.046
≥41	−0.023	−0.514	0.007	0.155	−0.019	−0.508
Years of service (years)						
≤ 5 (ref)						
6–10	−0.03	−1.299	0.104	4.162***	0.02	1.036*
11–20	−0.022	−0.717	0.107	3.221**	0.03	1.14
≥21	−0.016	−0.344	0.19	3.878***	0.076	1.979*
Professional title						
Junior (ref)						
Intermediate	0.013	0.697	0.019	0.888	0.022	1.366
Senior	0.003	0.147	0.028	1.414	0.016	1.043
Marital status						
Married (ref)						
Unmarried	−0.007	−0.371	−0.018	−0.875	−0.016	−0.975
Divorce or widowed	−0.003	−0.174	0.02	1.026	0.006	0.427
Education level						
Secondary school (ref)						
Junior college	−0.188	−1.843	−0.024	−0.216	−0.2	−2.297*
Undergraduate or above	−0.118	−1.156	0.027	0.24	−0.105	−1.21
Department						
Intensive care unit (ref)						
Medicine	−0.018	−0.548	−0.001	−0.026	−0.019	−0.66
Surgery	0.02	0.654	−0.049	−1.463	−0.003	−0.134
Obstetrics or gynecology	0.041	1.496	−0.025	−0.824	0.029	1.25
Pediatrics	0.009	0.372	0.004	0.156	0.011	0.533
Emergency	−0.025	−0.949	−0.021	−0.736	−0.036	−1.568
Medical technology department	−0.008	−0.299	−0.064	−2.144*	−0.039	−1.67
Others	0.051	2.124*	0.016	0.604	0.058	2.867**
Employment status						
Temporary (ref)						
Permanent	−0.026	−1.283	−0.088	−4.007***	−0.069	−3.963***
Hospital level						
Primary hospital (ref)						
Secondary hospital	−0.058	−1.673	0.042	1.11	−0.038	−1.28
Tertiary hospital	−0.102	−2.905**	−0.014	−0.364	−0.109	−3.637***
Constant	24.023	23.421***	37.637	7.147***	27.869	31.584***
R^2^	0.247		0.113		0.454	
F	34.939***		13.579***		85.044***	

*P < 0.05, **P < 0.01, ***P < 0.001.

**Table 4:** Direct and indirect effects of sense of security and its dimensions on turnover intention (*N* = 2480).

**Effect**	**B**	**BootSE**	**95%BootCI**	
			**Lower**	**Upper**
**Sense of security** ** → turnover intention**				
Total effect	−0.097	0.004	−0.105	−0.090
Direct effect	−0.067	0.005	−0.075	−0.058
Indirect effect	−0.031	0.002	−0.036	−0.026
**Environment** ** → turnover intention**				
Total effect	−0.444	0.020	−0.484	−0.405
Direct effect	−0.305	0.021	−0.346	−0.262
Indirect effect	−0.139	0.012	−0.162	−0.115
**Patient** ** → turnover intention**				
Total effect	−0.410	0.020	−0.450	−0.371
Direct effect	−0.276	0.021	−0.317	−0.234
Indirect effect	−0.134	0.012	−0.158	−0.111
**Self** ** → turnover intention**				
Total effect	−0.539	0.028	−0.595	−0.484
Direct effect	−0.345	0.029	−0.403	−0.286
Indirect effect	−0.195	0.017	−0.229	−0.163
**Organizational management** ** → turnover intention**				
Total effect	−0.275	0.012	−0.298	−0.252
Direct effect	−0.187	0.013	−0.212	−0.161
Indirect effect	−0.088	0.007	−0.102	−0.074
**Social support** ** → turnover intention**				
Total effect	−0.423	0.019	−0.461	−0.385
Direct effect	−0.287	0.021	−0.327	−0.246
Indirect effect	−0.135	0.012	−0.158	−0.113

## Discussion

This study constructed a hypothetical model based on theories to explore the relationship between nurses' sense of security and turnover intention during the normalization of the COVID-19 epidemic and with work engagement as a mediator in this relationship. We found that sense of security impacted positively and independently on work engagement and negatively and directly on turnover intention. Moreover, work engagement had a direct negative effect on turnover intention and partially mediated the relationship between sense of security and turnover intention among nurses. In addition, we examined the effects of the components of sense of security in this mediation model. All components of sense of security were negatively related to turnover intention. There were direct and indirect effects between the variables. It was shown that all components of sense of security (environment, patients, self, organizational management, and social support) may act as protective factors in reducing turnover intention and that work engagement partly mediated their relationship with turnover intention. This study may provide managers with new perspectives on sustaining the nursing team and developing nursing interventions during the normalization of the COVID-19 epidemic.

In the present research, the score of nurses' turnover intention was (14.13 ± 4.28), and nurses' turnover intention was at a high level during the normalization phase of the epidemic, which was higher than the findings of a previous survey of nurses in 23 hospitals in China before the epidemic (53). We revealed that more than half of the nurses reported high turnover intention (46.3%) or very high turnover intention (17.9%). This may result from the changes that the epidemic has brought to work. In order to control sporadic epidemics, nurses were required to work overtime and accepted emergency assignments to do large-scale screening, increasing psychological problems and burnout (45). One study noted that after the COVID-19 epidemic, the most reported predictors of nurses' turnover intention included fear of illness, stress, and anxiety (54). Meanwhile, the psychological impact of the epidemic may be long-lasting after the epidemic (55). All these reasons may lead to an increase in nurses' turnover intention.

Nurses' work engagement in this study was medium, consistent with previous studies under the COVID-19 epidemic (56, 57). During the normalized epidemic phase, despite the burnout of long-term epidemic prevention and control (58), the role played by nurses in the epidemic is socially recognized as high, which is conducive to nurses' professional identity (59). Higher work engagement could lead to higher self-efficacy and a sense of accomplishment in the workplace (60), leading to more inclination to stay with the organization. Consistent with previous literature, work engagement was negatively associated with turnover intention (61).

The study showed that nurses' sense of security tended to be on a high level, which indicates that nurses could feel supported and within control in the workplace during the epidemic normalization. The results showed that nurses' sense of security came most from organizational management, referring to hospital management and systems, leadership from superiors, and cooperation among colleagues, indicating that the participants indicated that the hospital was able to protect nurses' fundamental rights and interests (30). The excellent atmosphere and cohesive team were so that nurses could obtain support and security from the organization, consistent with the previous study (62, 63).

Our study found that nurses' sense of security had a direct negative predictive effect on turnover intention and an indirect negative predictive effect on turnover intention through work engagement. The sense of security is a subjective feeling in the workplace and is the basic need of clinical nurses. Therefore, in this work environment, nurses could be satisfied and benefit from their work (64). Furthermore, the five components of a sense of security were involved in the model. To some extent, work engagement partially mediated their association with turnover intention, indicating a positive process that reflected the JD-R model. It may be due to the open, inclusive and respectful work environment, nurse-patient relationship, and social support that allows nurses to speak up and generate positive creativity in their work, thus increasing their engagement and promoting commitment. In addition, in a well-managed organization, receiving recognition and praise from the team would provide positive feedback to promote engagement. When nurses feel protected by the organization, they feel satisfied with their work and are less likely to leave (65). The self-factor is the nurse's resilience and ability to face an adverse event that affects the individual's perception and behavior toward the event. It is worth noting that their direct effects are all greater than the indirect effects. In the current period, measures to enhance the sense of security may be more effective in reducing turnover intention. Therefore, it is recommended that an improved external environment and organizational support be considered in intervention programs to promote a sense of security and that the training of nurses is increased to improve competency. However, it is not enough to rely on hospital administrators alone; it is also necessary to improve the health care system and laws and regulations that protect the rights and interests of nurses. It is vital to increase the confidence and positive affect of nurses, which contributes to the professional identity of nurses and is very important to stabilize the nursing team and reduce nurse turnover.

We revealed the relationship between work engagement and turnover intention. Work engagement as a protective factor for turnover intention was consistent with previous studies (66, 67). The reason probably was that nurses with work engagement would benefit from their work. First, nurses with high work engagement are full of energy and can recover quickly, even in difficult situations (68). Secondly, nurses with high work engagement would have positive emotional and cognitive states and experience pleasure and immersion at work, as well as feel pride and inspiration at work (69). Work engagement as a positive resource facilitates the improvement of negative affect. Therefore, there is a suggestion that managers may facilitate nurses' work engagement and thus reduce turnover intention. In the context of the normalization of the COVID-19 epidemic, nurses were involved in the fight against the epidemic more extensively, and more nurses were involved. Providing nurses with support and rewards that match the effort promotes nurses' sense of belonging to organizations. At the same time, social media during that period also provided robust support for medical staff, contributing to increased work engagement.

The current study verified the relationship between work engagement and sense of security and turnover intention and the mediating role of work engagement in the relationship between sense of security and turnover intention. The findings of this study may have considerable clinical implications. The implications are far-reaching in the event of a significant public safety event or a major incident such as an infectious epidemic. They may threaten the sense of security of health care system professionals (70). The study was designed to enhance measures of sense of security and work engagement and may decrease nurses' turnover intention. It requires health systems, hospital administrators, and society to join together to increase investment in nursing and build a network of security to promote the sustainability of the nurse workforce. In addition, our study validated and supplemented JD-R Model and COR theory (42, 45). This study could be precious to fill the role of work engagement between sense of security and turnover intention in the Chinese context. Sense of security and work engagement as resources, primarily work engagement as an essential bridge between job resources and turnover intention, could help reduce turnover intention. The sense of security establishes an environment of mutual trust. A good sense of security would trigger individuals' tendency to avoid resource loss and motivation to create more work resources to dedicate more energy and effort input and increase their attachment to the organization, which provides a new perspective for reducing turnover intention.

## Conclusion

This study found that sense of security and work engagement were negatively associated with turnover intention. Work engagement mediated the relationship between sense of security and turnover intention. The results suggest that managers should formulate policies and strategies to ensure and improve the interests and well-being of nurses and improve the practice environment to protect the sense of security of nurses, which is helpful to increase work engagement and reduce turnover intention. In addition, formulating strategies and measures to encourage and reward nurses in the work of expression and innovation, and improve work participation, will also help reduce the intention to leave, reduce the occurrence of turnover, and maintain the stability of the nurse team.

## Limitation

Firstly, this study used a convenience sampling method considered simple, applicable, and appropriate for the study purpose. However, this method has the potential to produce sampling bias. Secondly, the subjects of this study are all nurses from hospitals at all levels in various regions of guangdong province, which can only represent the level of sense of security, work engagement, and turnover intention among nurses in Guangdong Province. Guangdong Province is a relatively developed province in China, and economic development may cause differences in different regions. Third, this sample was collected from September 2020 to May 2021, which may be biased. Finally, as this study was a cross-sectional survey, it cannot explain the causal relationship between relevant factors and turnover intention.

## Data availability statement

The original contributions presented in the study are included in the article/Supplementary material, further inquiries can be directed to the corresponding author.

## Ethics statement

The studies involving human participants were reviewed and approved by the Ethics Committee of Guangdong Provincial People's Hospital (No. Ky2020-579-01). The patients/participants provided their written informed consent to participate in this study.

## Author contributions

H-gH: conceptualization. Q-xH: methodology. YT: software, formal analysis, and original draft preparation. S-bW and LD: writing—review and editing. All authors contributed to the article and approved the submitted version.

## Funding

This work was supported by the Guangdong Basic and Applied Basic Research Foundation under (Grant No. 2021A1515012187), the Guangdong Health Economics Association Key Projects under (Grant No. 2022-WJZD-01), and Guangdong Provincial People's Hospital, Guangdong Academy of Medical Sciences Project under (Grant No. 7197040908).

## Conflict of interest

The authors declare that the research was conducted in the absence of any commercial or financial relationships that could be construed as a potential conflict of interest.

## Publisher's note

All claims expressed in this article are solely those of the authors and do not necessarily represent those of their affiliated organizations, or those of the publisher, the editors and the reviewers. Any product that may be evaluated in this article, or claim that may be made by its manufacturer, is not guaranteed or endorsed by the publisher.
